# Effect of Nanoparticles Exposure on Fractional Exhaled Nitric Oxide (FENO) in Workers Exposed to Nanomaterials

**DOI:** 10.3390/ijms15010878

**Published:** 2014-01-09

**Authors:** Wei-Te Wu, Hui-Yi Liao, Yu-Teh Chung, Wan-Fen Li, Tsui-Chun Tsou, Lih-Ann Li, Ming-Hsiu Lin, Jiune-Jye Ho, Trong-Neng Wu, Saou-Hsing Liou

**Affiliations:** 1 Division of Environmental Health and Occupational Medicine, National Health Research Institutes, Miaoli 350, Taiwan; E-Mails: ader.una@gmail.com (W.-T.W.); bobbibrownq@nhri.org.tw (H.-Y.L.); 010521@nhri.org.tw (Y.-T.C.); tctsou@nhri.org.tw (T.-C.T.); lihann@nhri.org.tw (L.-A.L.); tnwu@mail.cmu.edu.tw (T.-N.W.); 2 Division of Medical Genetics, Department of Medicine, University of Washington, Seattle, WA 98195, USA; E-Mail: wanfenli@u.washington.edu; 3 Institute of Occupational Safety and Health, Council of Labor Affairs, Executive Yuan R.O.C., New Taipei 221, Taiwan; E-Mails: mhlin@mail.iosh.gov.tw (M.-H.L.); hjj@mail.iosh.gov.tw (J.-J.H.); 4 Graduate Institute of Biostatistics, China Medical University, Taichung 402, Taiwan; 5 Department of Public Health, National Defense Medical Center, Taipei 114, Taiwan; 6 Department of Public Health, College of Public Health, China Medical University, Taichung 402, Taiwan

**Keywords:** nanoparticles, nanomaterials, workers’s respiratory health, airway inflammation, Fractional exhaled nitric oxide, occupational epidemiology

## Abstract

Fractional exhaled nitric oxide (FENO) measurement is a useful diagnostic test of airway inflammation. However, there have been few studies of FENO in workers exposed to nanomaterials. The purpose of this study was to examine the effect of nanoparticle (NP) exposure on FENO and to assess whether the FENO is increased in workers exposed to nanomaterials (NM). In this study, both exposed workers and non-exposed controls were recruited from NM handling plants in Taiwan. A total of 437 subjects (exposed group = 241, non-exposed group = 196) completed the FENO and spirometric measurements from 2009–2011. The authors used a control-banding (CB) matrix to categorize the risk level of each participant. In a multivariate linear regression analysis, this study found a significant association between risk level 2 of NP exposure and FENO. Furthermore, asthma, allergic rhinitis, peak expiratory flow rate (PEFR), and NF-κB were also significantly associated with FENO. When the multivariate logistic regression model was adjusted for confounders, nano-TiO_2_ in all of the NM exposed categories had a significantly increased risk in FENO > 35 ppb. This study found associations between the risk level of NP exposure and FENO (particularly noteworthy for Nano-TiO_2_). Monitoring FENO in the lung could open up a window into the role nitric oxide (NO) may play in pathogenesis.

## 1. Introduction

Many countries are currently supporting nanotechnology research and development for the production of nanomaterials (NM) due to the considerable economic potential of the technology. The market for NM is increasing rapidly and is predicted to have a $3.1 trillion impact on the global economy by 2015 [1]. Although nanotechnology is applied to many different domains, engineered NM produced and handled in industrial and academic settings present new challenges in managing potential health risks to workers, consumers, and the environment [2,3]. Until now, however, most of the documents about the health hazards of nanoparticles (NP) have been provided mainly from animal or *in vitro* studies. The epidemiological data available on nanometric particles refer to environmental ultrafine particles (UFPs) while no data are available for NP exposed workers [4,5]. In most studies described above, these results suggested that UFP may be more toxic than particulate matter (PM) ≤ 2.5 and indicate an adverse relationship on cardiovascular and pulmonary morbidity and mortality; however, results are not consistent [4,5]. In addition, the physical and chemical characterization between engineered NP and ambient UFPs was totally different [5,6], and it may lead to inconsistencies in health hazards.

Previous studies also showed that NP mainly deposit (75%–80%) in the alveolar region where particles interfere with or within cells (like epithelial cells and macrophages) as well as with the mucus and clearance of NP from the lung, is slower than that of fine particles (PM ≤ 2.5) [2,7–9]. Animal studies mentioned that the greater surface area per mass of NP is more active biologically than larger-sized particles with the same chemistry, and that particle surface area and number appear to be better predictors for NP-induced inflammatory and oxidative stress responses in the lung [9–11]. The number of workers who deal with NM is increasing rapidly; therefore, the validation methods to evaluate the inhalation toxicity of engineered NM in human are really needed.

The field of fractional exhaled nitric oxide (FENO) measurement has developed rapidly in recent decades. Measurement of FENO produced by the human lung and present in the exhaled breath is now recognized as a safe and useful diagnostic test of airway inflammation [12]. The European Respiratory Society (ERS) and the American Thoracic Society (ATS) have provided evidence that FENO is elevated in many lung diseases including asthma, atopy, upper airway viral infections, post-transplant lung rejection, radiation pneumonitis, and fibrosing alveolitis [12,13]. Furthermore, numerous studies have provided evidence regarding the applications of FENO in exposure assessment for epidemiologic studies [14,15]. Based on these studies, the results showed that the length of roads—traffic pollution—was positively associated with FENO in children with asthma [14], and that short-term increases in community-level ambient PM2.5 and PM10 were associated with elevated FENO [15]. To date, almost no research has been done on values and determinants of FENO in workers exposed to NM.

Therefore, the purpose of the study was to examine the effect of NP exposure on FENO and to investigate the determinants of increased FENO in workers exposed to NM.

## 2. Results

### 2.1. Participant Characteristics and FENO Values

Table 1 shows that FENO levels were significantly elevated in workers who were male, never smoked, and in the RL2 group. The results also found that workers who exercised 3 h before the tests had a decreased level in FENO (13.3 ± 8.5 *vs*. 21.0 ± 17.5 ppb; *p* = 0.008). The FENO levels were compared between workers with and without different kinds of diseases; we found that workers with asthma or allergic rhinitis had significantly increased FENO compared to those without these diseases.

### 2.2. Association of Determinants with FENO Values

Significantly positive associations appeared between FENO and height (*r* = 0.098, *p* = 0.041), body weight (*r* = 0.105, *p* = 0.028), LnNF-κB (*r* = 0.188, *p* <0.001), and PERF (*r* = 0.098, *p* = 0.041) among all participants (Table 2).

After all variables were included in the multivariate regression analysis with the natural logarithm for FENO, we found that the NP exposed RL2 group had higher FENO levels than the control group (Table 3). Furthermore, cigarette smoking, exercise before tests, asthma, allergic rhinitis, PEFR (percentage), and NF-κB (EBC) were significant variables for FENO. Stepwise multiple regressions were used to choose the predictor variables carried out by the automatic procedure. This model presented the same significant variables for FENO except gender.

FENO values were further examined in different NM exposed categories. After adjusting for gender (male *vs*. female), cigarette smoking (yes *vs*. no), exercise before tests (yes *vs*. no), asthma (yes *vs*. no), allergic rhinitis (yes *vs*. no), LnNF-κB (EBC), and PEFR (%), it was found that compared to the control group, Nano-TiO_2_ exposed group had significantly higher FENO levels, regardless of using the enter or stepwise methods (Table 4).

### 2.3. Risk Levels in FENO Values

**Figure 1.**
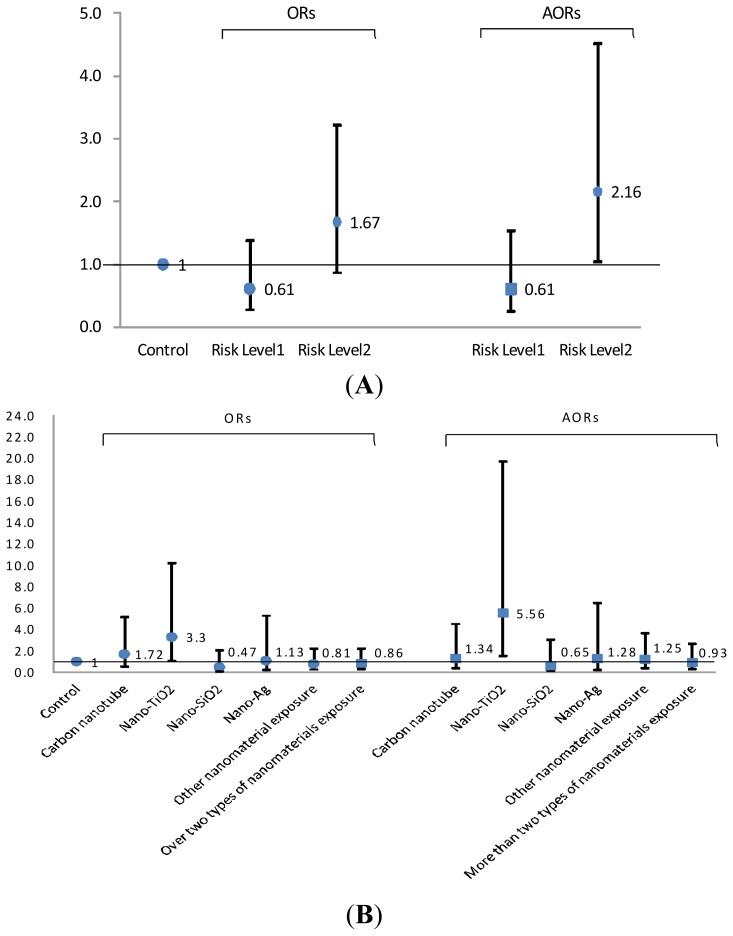
The odds ratio of risk levels (**A**) and by nanomaterials exposure (**B**) in fractional exhaled nitric oxide (FENO) values > 90th percentile (35 ppb) of all 437 study subjects. [AOR: adjusted odds ratios for gender (male *vs*. female), cigarette smoking (yes *vs*. no), exercise before tests (yes *vs*. no), asthma (yes *vs*. no), allergic rhinitis (yes *vs*. no), LnNF-κB (EBC), and PEFR (%)].

In Figure 1, a multivariate logistic regression model was used to assess the association of risk levels in FENO values >35 ppb of all included subjects. When this model was adjusted for gender, cigarette smoking, exercise before tests, asthma, allergic rhinitis, NF-κB (EBC), and PEFR (%), the RL2 group and the Nano-TiO_2_ exposure group showed significantly increased risk in FENO in comparison to the control group (AORs: 2.16 and 5.56; 95% CI: 1.03–4.51 and 1.57–19.72, respectively). Similar associations for cigarette smoking, exercise before tests, asthma, allergic rhinitis, PEFR (percentage), LnNF-κB (EBC) were still observed in this model, although the data are not shown. Only Nano-TiO_2_ exposed and control groups (*n* = 213) were selected to calculate the risk in FENO > 35 ppb in a multivariate logistic regression model that adjusted for the same variable.

## 3. Discussion

For this study, the workers exposed to NM with a nano-tool risk level matrix greater than the second level had significantly higher FENO levels compared with the control group. In all of the NM exposed categories, nano-TiO_2_ was particularly noteworthy, because it had a significantly increased risk in FENO. Significant associations for FENO were also observed in such variables as gender, cigarette smoking, asthma, allergic rhinitis, replicating findings in other studies. Furthermore, the results also showed that NF-κB and PEFR were associated with increased FENO.

### 3.1. Inflammation and NOS2 Expression

Inhalation of NP is the mechanism most widely researched and the interaction of NP with epithelial cells in the lungs is of interest. NP can be deposited in the respiratory tract, where they have been associated with oxidative stress related inflammatory reactions and damage to epithelial cells from reactive oxygen species and activation of regulation factors [2,7,8,16]. Nitric oxide (NO) is a gaseous signaling molecule that is generated by three isoenzymes of NO synthase (NOS) that are differentially regulated and expressed in the airways. The three isoenzymes of NOS also appear to play different pathophysiologic roles [13,17]. Among those, inducible NOS (NOS2) is constitutively expressed in the human airway epithelium, but its expression can be increased many times by inflammatory agents on macrophages. Furthermore, neuronal NOS (NOS1) and endothelial NOS (NOS3) are constitutively expressed enzymes in the lung that produce NO in low amounts and have an absolute requirement for intracellular calcium/calmodulin. The previous study showed that FENO variability is largely determined by epithelial NOS2 expression, with little contribution from other isoforms [18]. This study supports the hypothesis that NP enter in the respiratory tract where they produce inflammatory reactions to induce NOS2 expression that can be monitored in the exhaled breath.

### 3.2. Different Nanomaterials (NM) Exposure and NOS Expression

Until now, there has been little research of FENO in workers exposed to NM. According to past *in vitro* and *in vivo* studies, exposure to TiO_2_ NP increases the NO in the mouse’s brain after intragastric administration with TiO_2_ NP for 60 consecutive days. After TiO_2_ NP enter the mouse’s brain, the excitatory neurotransmitter-Glu in the brains was significantly increased and the binding of Glu and NMDA receptors can activate calcium-dependent protease, *i.e*., NOS [19]. Another *in vitro* study showed that the NO production was only increased by exposure to TiO_2_ NP in human umbilical vein endothelial cells (HUVECs), but the effect of the NO-dependent vasodilatory function was not observed [20]. Moreover, the increased NO content may arise from NOS2 activity, which has been upregulated in HUVECs during inflammation reactions. These three studies show that microvascular dysfunction associated with exposure to TiO_2_ NP is not due to altered arteriolar smooth muscle responsiveness of NO [21–23], however they did not detect whether TiO_2_ NP produced inflammatory reactions induce epithelial NOS2 expression.

Other NM exposure studies found that rat coronary endothelial cells exposed for 24 h to high doses of Ag NP (45 nm) (100 μg/mL) induce NO-dependent proliferation through activation of endothelial nitric oxide synthase (NOS3) [24], but this was not found at lower doses (<10 μg/mL). In another study, thirty days after exposure of silica NP (600 μg/rat), the results showed that pulmonary NOS2 was not enhanced in any of the evaluated endpoints [25]. In a study of iron oxide NP treatments in the human aortic endothelial cells (HAECs), the results showed that the NOS activity and NO levels were significantly elevated at relatively low doses of iron oxide NP (Fe_2_O_3_ and Fe_3_O_4_) [26], but the same result was not found in this study.

### 3.3. Determinants of Exhaled Nitric Oxide Levels (FENO)

This study found that gender, cigarette smoking, asthma, and allergic rhinitis were significant variables for FENO. The ATS guidelines on the use of FENO mentioned that factors such as sex, asthma, atopy, and current cigarette smoking need to be taken into account when predicted values for FENO are derived from population-based reference equations [13,17]. Gender-related differences are biologically plausible as several *in vitro* studies have suggested that estrogen affects the expression of NOS [27], and therefore, may influence NO flux from the airway epithelium. In the majority of studies, FENO levels in males were higher than in females, when controlling for height, weight, lung function, smoking, atopy, asthma and rhinitis [17,28].

Previous studies also report that cigarette smoke decreases expression of inducible nitric oxide synthase (iNOS) and thus the interpretation of a FENO result obtained from a current smoker should be done with caution [12,13,17,29,30]. The possible explanation could be related to the smoking-induced high levels of exogenous NO and that this depends on a down-regulation of endogenous NO, such as NOS2 in lung epithelial cells and NOS3 in pulmonary artery endothelial cells [31–33]. Decreased endogenous NO may also be a result of reduced ciliary activity and local clearance from the airways [29]. Moreover, previous studies indicated that FENO levels increased in subjects after they quit smoking, but former smokers still had lower FENO levels compared with healthy control subjects [34,35]. The results showed that long-term cigarette smoking is associated with permanent reductions in FENO in smokers, even if they quit smoking.

Although the guideline of ATS mentioned that on the implementation of FENO it would seem prudent to avoid strenuous exercise before the measurement [36], the mechanism between exercise and FENO is still not clear. Our results regarding the effect of exercise on FENO are consistent with earlier studies [37,38]. As stated, these studies showed a drop in FENO values after exercise both in asthmatic and healthy children [37,38]. We thought that changes in bronchial diameter may be responsible for reduced FENO levels after exercise. The reduced airway surface area might have led to lower NO diffusion through the airways when using a constant exhalation flow during FENO measurements.

### 3.4. NF-κB in Exhaled Breath Condensate

Biomarkers in EBC can directly reflect airway inflammation and oxidative stress. In this study, the concentration of NF-κB in exhaled breath condensate was positively correlated with FENO. Previous studies showed an increased expression of NOS2 in airway epithelial cells, likely to be due to increased transcription mediated via the transcription factors NF-κB [39]. Activation of NF-κB triggers the expression of inflammatory cytokines. Consequently, polymorphonuclear leukocytes are attracted, activated, and result in NOS2 [39,40].

### 3.5. Strengths and Limitations

This study has several strengths. To the best of our knowledge, this study of the association between the many indicators of NM exposure and FENO in workers exposed to NM is the only one to date. Moreover, the authors adopted a comprehensive study design to validate the values and determinants of FENO among workers exposed to NM, in the ongoing occupational cohort study from 14 NM-handling plants in Taiwan. The common determinants of FENO such as age, gender, height, atopy, variability in test time (the routine health check-up was taken in the morning), smoking, exercise before tests, and diet (fasting before routine health check-up) were considered.

There are some limitations to this study. First, a lack of personal sampling resulted in us being unable to analyze the realistic dose-response relationship. Workers involved in the handling of engineered nanomaterials are most probably exposed by inhalation; however, published information on exposures in the workplace is sparse. The main reason for this sparseness is that measuring exposure to engineered nanoparticles is not an easy task [41–45]. The behaviors and characteristics of engineered nanoparticles differ in several ways from traditional aerosols [9,46]. There is still insufficient scientific evidence to decide on which particle size range and exposure parameters of engineered nanoparticles should be measured to characterize exposure, or which are the most appropriate instruments or methods to be used [41–45]. Faced with uncertainties relating to nanomaterial exposure assessment, control banding principles have recently become popular [47,48]. With the control banding approach, hazard (severity) bands are generated based on toxicologic data of nanomaterials combined with exposure (probability) bands reflecting the exposure levels. Control banding is a semi-quantitative assessment of the risk that offers the minimal preventive measures to be implemented according to the estimated level of risk. Second, the heterogeneity of NM made it difficult to find a sufficiently large group of workers exposed to the same particles and to present potential health effects of any one NM. In spite of this, this study still found that only workers exposed to nano-TiO_2_ had a significant increase in FENO. Thirdly, genetic polymorphisms of NOS may be related to FENO levels, but until now the results have been inconsistent.

## 4. Subjects and Methods

### 4.1. Study Subjects and Data Collection

We conducted a survey of the nanotechnology plants in Taiwan. According to the lists of NM handling plants from the Environmental Health and Safety project, this study excluded some which were selling only, but not handling raw NM, some which were shut-down, or had never used NM, or were not currently using NM. The basic information on these factories that agreed to participate in this study is listed in Table S1. Among these 13 factories, five factories manufactured NM and 12 factories applied NP to manufacture other products. The physico-chemical properties of NM manufactured and/or used in these factories are listed in Table S2. These factories hired 515 workers from2009–2011, and the participation rate in this study was 89% (458/515).

The judgment of exposed workers and non-exposed controls was based on an industrial hygienist and supervisor in each factory. To ensure correct classification, we also requested workers and the company to provide NM category, size and amount in the handle process. The collection of the above information had been added in Table S3. The non-exposed controls were selected from workers at the same plants as the exposed workers, but who did not handle NM. Two hundred and fifty eight workers exposed to NM and 200 non-exposed controls were recruited to take part in this study from 2009–2011. The Institutional Review Board of National Health Research Institutes, Miaoli, Taiwan, approved this study. Informed consent was obtained from each of the subjects after a detailed explanation of the nature and possible consequences of the study by the interviewer on the day of personal interview. After a written informed consent was obtained from individual participants, the subjects were interviewed in person using a structured questionnaire and health examinations. FENO measurements were taken, spirometry was conducted, and each person was interviewed using a structured questionnaire. Information was collected on age, gender, exercise, smoking and alcohol consumption, history of disease, NM handling, and exposure probability at the workplace.

FENO measurements were performed on workers when they received their routine health check-up on one morning per year. Lung function measurements were taken after the FENO measurement. For each participant, the authors also collected exhaled breath condensate (EBC) and stored it at −80 °C, and subsequently measured nuclear factor kappa B (NF-κB). A total of 437 subjects (exposed group = 241, non-exposed group = 196) completed the measurements. The complete examination rate was 95%.

### 4.2. Fractional Exhaled Nitric Oxide (FENO) and Pulmonary Function Measurement

A chemiluminescence analyzer (Bedfont Scientific, Kent, UK) was used according to current guidelines of the ATS/ERS to measure FENO. The chemiluminescence analyzer process was repeated at least three times to ensure reproducibility (correlation coefficient is 0.960, *p* < 0.001). Exhalation time for each subject was 12 s with a flow of 50 mL/s.

Subjects tested with the computerized spirometer (Chest graph HI-701, Chest M.I. Inc. Hongo, Bunkyo-Ku-Tokyo, Japan). The spirometer directly enters age, gender, height, and race, in order to calculate the predicted normal lung function value and to determine the percentage of the predicted value. The spirometer calibration check was performed by a 3-liter-calibration pump before each testing session. The lung function parameters examined forced expiratory volume at 1 s (FEV1), forced vital capacity (FVC), maximum mid-expiratory flow (MMF), peak expiratory flow rate (PEFR), the FEV1/FVC ratio, and forced expiratory flow (FEF) between 25% and 75% of FVC.

### 4.3. Exhaled Breath Condensate (EBC) Collection

EBC samples were collected during the 15 minutes of tidal breathing by using a single-use disposable collecting circuit (DECCS 04 ST, Medivac, Parma, Italy) with an ECoScreen Turbo (VIASYS Healthcare GmbH, Hoechberg, Germany). The temperature of −6 °C was constantly maintained during the collection. EBC samples were stored at −80 °C in polypropylene tubes until assayed.

### 4.4. Nuclear Factor Kappa B (NF-κB) Assay

The luciferase reporter assays (inflammatory response reporter array) were used to detect the activation of inflammatory response transcription factors. HL-CZ cells were cultured overnight on 96 well plate (total volume = 50 μL cell suspension/well, 1 × 10^4^ cells/well in triplicate). Then to every well was added 50 μL pAC NF-κB (MOI = 0.2) for 16 h, 100 μL TNF-α (200 pg/mL) and 10 μL EBC sample for 6 h of incubation. Cell lysates were collected using 60 μL of lysis buffer (0.1 M KH2PO4, pH 7.9, 0.5% Triton X-100, 1 mM DTT). Luciferase activity was measured by standard protocols [49].

### 4.5. Exposure Assessment

Methods for measuring occupational exposure need to be developed in order to identify and monitor hazards associated with NP exposure and to provide workers with recommendations for reducing exposures [4,5,41,50,51]. However, there is still no standardized method of monitoring exposure to NM in the field of occupational health. Only limited research into monitoring of exposure has been conducted due to the instrument barrier [44,45,52–55], and many real-world results have shown the levels of exposure to be likely transient or very low.

In such a context of uncertainty in exposure assessment, the control banding (CB) approach may be helpful in implementing a risk-management strategy according to a precautionary approach. Recently, worldwide several CB approaches for manufactured NM-related exposure have been developed and published [47,56–61]. Despite limitations, in the absence of occupational exposure limits (OELs), CB may be a useful strategy for assessing and controlling occupational hazards as part of a comprehensive safety and health program [61].

In this study, the CB nano-tool risk level matrix proposed by Dr. Paik and his colleagues was used to categorize the risk level of each participant [47]. We used the latest version of “CB Nanotool 2.0” [62]. Briefly, the risk level matrix was calculated based on the severity score of the NM toxicity and the score of the exposure probability. The factors considered in the calculation of the severity score included NM (70% of severity score) and parent material (30% of severity score). In order to obtain consistent scores, the NM toxicity severity score was based on the summary tables from the review document. The exposure probability score was based on the questionnaires collected from individual workers exposed to the various NM. The cross-table of the severity scores and probability scores were used to generate the risk levels (RLs, 1–4) for each individual. The higher the risk level, the higher the severity of NM toxicity and/or the higher the exposure probability. The detailed information of the summary of the most important characteristics of probability scores, severity factors and scores, and CB nano-tool risk level matrix are presented in Tables S4 and S5. The comparison with the classification of the different NPs in terms of Risk levels are shown in Table S6.

### 4.6. Statistical Analysis

The FENO and NF-κB exhibited right-skewed distributions. Therefore the natural logarithmic transformation was applied. Means and standard deviations were used to describe the distributions of continuous variables. The Student’s *t*-test and analysis of variance were used to test the differences among risk variables. Pearson’s correlation analysis was used to measure the association for continuous variables. A linear regression model and a logistic regression model were used to compare the FENO between different risk level (RL) workers and the controls, based on the risk levels categorized from the CB method mentioned above. The workers were divided into seven groups depending on whether the workers handled and used NM in the workplace: (1) control group; (2) carbon nanotube exposed group; (3) nano-TiO_2_ exposed group; (4) nano-SiO_2_ exposed group; (5) nano-Ag exposed group; (6) other NM exposed group; and (7) more than two types of NM exposed group. Based on the published literature [63,64], these studies suggested that a FENO value greater than 35 ppb was predictive of poor asthma control. Moreover, FENO levels of <35 ppb are accepted as normal values for healthy adults when measured by standard methods in different populations [65]. In our study subjects, the 90th percentiles of FENO values also were 35 ppb. Thus we selected 35 ppb as a cutoff point for high and low NO in this study. The statistical analyses were performed using SPSS, version 19.0 for Windows. All statistical tests were two-sided with *p* < 0.05 as the level of statistical significance.

## 5. Conclusions

Until recently, scientists had little information regarding the health hazards of NP exposure. Employee medical surveillance is a strategy for providing benefits to the individual and company for health outcomes for workers exposed to NM. Among the health checkups, FENO might be a useful indicator of broader epithelial function in addition to being an inflammatory marker for workers potentially exposed to NP, although this aspect requires more investigation. Therefore, monitoring FENO in the lung could open up a window into the role NO may play in pathogenesis.

## Supplementary Information



## Figures and Tables

**Table 1.** Demographic characteristics and fractional exhaled nitric oxide (FENO) values of study participants (*n* = 437).

Variables	All subjects (*n* = 437)			

	Exhaled nitric oxide (ppb)			*p*-Value a

	*n*	Mean	(SD)	
Age				0.650
≤40 years	317	20.7	(17.8)	
>40 years	120	20.2	(15.5)	
Gender				0.011
Male	300	21.7	(18.0)	
Female	137	18.0	(15.0)	
Ethnic groups				0.084
Hoklo	341	19.8	(15.9)	
Hakka	55	26.6	(25.6)	
Others	39	19.4	(12.1)	
Education				0.253
≤Senior high and vocational school	68	16.5	(8.6)	
University and College	206	21.3	(18.5)	
≥Graduate School	159	21.4	(17.9)	
Cigarette smoking				0.011
Current smokers	66	15.5	(10.6)	
Never smokers	368	21.4	(17.9)	
Alcohol use				0.998
Yes	40	19.0	(12.2)	
No	396	20.8	(17.6)	
Exercise before tests				0.008
Yes	24	13.3	(8.5)	
No	410	21.0	(17.5)	
Risk levels				0.040
Control	196	20.5	(19.1)	
Risk level 1	126	18.1	(12.2)	
Risk level 2	115	23.3	(18.2)	
Control and nanomaterial exposed groups				0.068
Control	196	20.5	(19.1)	
Carbon nanotube	57	24.1	(18.7)	
Nano-TiO_2_	17	28.0	(21.5)	
Nano-SiO_2_	36	17.9	(10.8)	
Nano-Ag	16	20.8	(11.3)	
Other NM exposure	54	16.5	(10.6)	
More than two types of NM exposure	61	20.3	(16.3)	
**Disease History**				
Chronic bronchitis				0.139
Yes	23	25.8	(20.1)	
No	412	20.3	(17.0)	
Asthma				<0.001
Yes	9	58.7	(37.8)	
No	425	19.8	(15.6)	
Allergic rhinitis				<0.001
Yes	80	27.1	(22.8)	
No	355	19.2	(15.4)	
Atopic dermatitis				0.257
Yes	27	24.2	(15.6)	
No	409	20.4	(17.3)	
Hypertension				0.731
Yes	31	19.1	(10.3)	
No	402	20.7	(17.7)	

a *t*-test to assess the difference in mean natural log(ln)-transformed FENO.

**Table 2.** Correlation matrix of LnFENO, age, height, weight, LnNF-κB, and Pulmonary function (*n* = 437).

	LnFENO	Age (years)	Height (cm)	Weight (kg)	LnNF-κB (EBC)
Age (years)	0.033	1			
Height (cm)	0.098 *	−0.231 **	1		
Weight (kg)	0.105 *	−0.083	0.631 **	1	
LnNF-κB (EBC)	0.188 **	0.031	0.141 **	0.126 **	1
FEV1.0%	0.028	−0.240 **	−0.100 *	−0.207 **	−0.006
FVC (%)	−0.048	0.000	0.141 **	0.117 *	−0.058
MMF (%)	−0.021	−0.052	−0.086	−0.107 *	−0.059
PEFR (%)	0.098 *	0.048	−0.022	0.065	−0.048
FEF25 (%)	0.056	−0.005	0.021	0.082	−0.048
FEF50 (%)	−0.035	−0.073	−0.075	−0.037	−0.069
FEF75 (%)	−0.036	−0.149 **	0.014	−0.138 **	−0.046
FEV1/FVC	0.012	0.285 **	−0.215 **	−0.168 **	−0.061

* 0.01 < *p* < 0.05;

** *p* < 0.01.

NF-κB (EBC) indicates NF-κB in exhaled breath condensate; FEV1, forced expiratory volume at 1 second; FVC, forced vital capacity; MMF, maximum mid-expiratory flow; PEFR, peak expiratory flow rate; FEF25, forced expiratory flow at 25%; FEF50, forced expiratory flow at 50%; FEF75, forced expiratory flow at 75%; FEV1/FVC, the FEV1/FVC ratio.

**Table 3.** Multiple linear regression model for determinants of fractional exhaled nitric oxide (FENO) values a.

	Mode1 (Enter)			Mode2 (Stepwise)		
		
	β	SE	*p*-Value	β	SE	*p*-Value
Risk levels						
RL1 *vs*. control	−0.050	0.076	0.506	−0.056	0.075	0.459
RL2 *vs*. control	0.178	0.078	0.023	0.170	0.077	0.028
Age (years)	0.006	0.004	0.143			
Gender (Male *vs*. Female)	0.118	0.102	0.250	0.198	0.071	0.006
Height (cm)	0.006	0.006	0.327			
Weight (kg)	0.001	0.003	0.836			
Cigarette smoking (Yes *vs*. No)	−0.237	0.093	0.011	−0.233	0.093	0.012
Exercise before tests (Yes *vs*. No)	−0.296	0.139	0.034	−0.295	0.138	0.034
Asthma (Yes *vs*. No)	1.020	0.236	<0.001	1.042	0.222	<0.001
Allergic rhinitis (Yes *vs*. No)	0.289	0.081	<0.001	0.289	0.080	<0.001
PEFR (%)	0.006	0.002	0.001	0.006	0.002	0.001
LnNF-κB (EBC)	0.204	0.069	0.003	0.213	0.068	0.002

a Using multiple linear regression models to relate natural log(ln)-transformed FENO.

**Table 4.** Correlation of determinants of fractional exhaled nitric oxide (FENO) values in different nanomaterial exposed categories a.

	Mode1 (Enter)			Mode2 (Stepwise)		
		
	β	SE	*p*-Value	β	SE	*p*-Value
Nanomaterials exposure						
Carbon nanotube *vs*. control	0.045	0.124	0.715	0.030	0.122	0.807
Nano-TiO_2_ *vs*. control	0.351	0.166	0.035	0.334	0.165	0.044
Nano-SiO_2_ *vs*. control	0.007	0.120	0.956	0.022	0.119	0.857
Nano-Ag *vs*. control	0.153	0.170	0.367	0.127	0.169	0.452
Other NM exposure *vs.* control	−0.039	0.100	0.695	−0.049	0.100	0.623
More than two types of NM exposure *vs*. control	0.051	0.098	0.600	0.052	0.097	0.591
Age (years)	0.006	0.004	0.131			
Gender (Male *vs*. Female)	0.099	0.104	0.342	0.182	0.073	0.013
Height (cm)	0.005	0.006	0.425			
Weight (kg)	0.002	0.003	0.625			
Cigarette smoking (Yes *vs*. No)	−0.222	0.094	0.018	−0.219	0.093	0.019
Exercise before tests (Yes *vs*. No)	−0.275	0.141	0.051	−0.274	0.140	0.051
Asthma (Yes *vs*. No)	1.008	0.239	<0.001	1.053	0.225	<0.001
Allergic rhinitis (Yes *vs*. No)	0.289	0.082	<0.001	0.290	0.081	<0.001
PEFR (%)	0.006	0.002	0.002	0.006	0.002	0.001
LnNF-κB (EBC)	0.185	0.070	0.009	0.196	0.069	0.005

a Using multiple linear regression models to relate natural log (ln)-transformed FENO.
