# Supplementary material for: Effect of Nanoparticles Exposure on Fractional Exhaled Nitric Oxide (FENO) in Workers Exposed to Nanomaterials
Source: Int J Mol Sci. 2014 Jan 9;15(1):878–94. doi: 10.3390/ijms15010878 (PMC3907844; doi:10.3390/ijms15010878)
Supplement: Supplementary file 1 [file ijms-15-00878-s001.pdf]

Supplementary Information

Table S1. The basic hygiene information of 13 factories participated in this study.

Factory number	Industry	Starting year	Workplace	Dust control method	Handling of Leakage
1	Photocatalyst mfg	2006	Enclosed	Local ventilation	Sweeping
2	Nanomaterials mfg	2004	Enclosed	Local ventilation	Flushing with water
3	Nanomaterials mfg	2007	Open	Local ventilation	Flushing with water
4	Toilet mfg	2001	Enclosed	Local ventilation	Flushing with water
5	Air cleaner mfg	2004	Open	Local ventilation and General ventilation	Sweeping
6	Toilet mfg	2007	Enclosed	Local ventilation and General ventilation	Flushing with water
7	LED mfg	2004	Open with hood	Local ventilation and General ventilation	Sweeping
8	LED mfg	2002	Enclosed	Local ventilation and General ventilation	Flushing with water
9	Paint mfg	2001	Open	Local ventilation and General ventilation	Sweeping
10	Colorants mfg	2003	Enclosed	Local ventilation	Sweeping
11	Carbon nanotube mfg	2004	Open	Local ventilation	Vacuum cleaner or sweeping
12	Textile mfg	2006	Enclosed	Local ventilation	Sweeping
13	Self-cleaning tiles mfg	2008	Open	General ventilation	Flushing with water

Abbreviation: mfg: manufacturing; LED: light emission device.

Table S2. The characteristics of nanomaterials used or manufactured in these 13 factories.

Factory number	Type of nanomaterial handling	Nanomaterial used/Mfd	Major nanomaterial used/Mfd	Size (nm)	Amount used/Mfd (mg/Time)	Duration of use/Mfg (Hour/Time)	Frequency of use/Mfg (Times/Week)	Type of nanomaterials used/Mfd
1	Use	Nano-silver	Nano-silver	Unknown	20	5	5	Liquid solution
2	Use	Fe_2O_3	Fe_2O_3	6–10	5000	2	1	Liquid solution
		Nano-gold		3–40	19	0.1	1	Liquid solution
		Nano-silver		5–10	2.1	0.1	1	Liquid solution
	Mfg	Fe_2O_3	Fe_2O_3	6–10	5000	0.2	8	Liquid solution
		Nano-gold		3–40	10	0.25	8	Liquid solution
		Nano-silver		5–10	21	0.1	8	Liquid solution
3	Use	Titanium dioxide	Titanium dioxide	15–20	10,000	2.5	2.5	Powder and liquid solution
	Mfg	Titanium dioxide	Titanium dioxide	15–20	1,000,000	1	8	Powder and liquid solution
4	Use	Nano-silver	Nano-silver	Commercial secret	Commercial secret	3	7	Liquid solution
	Mfg	Nano-silver	Nano-silver	Commercial secret	Commercial secret	7	3	Liquid solution
5	Use	Titanium dioxide	Titanium dioxide	20	50,000	4	1	Liquid solution
6	Use	Silicon dioxide	Silicon dioxide	10	50,000	1	1	Liquid solution
		Nano-silver		100	50	1	1	Liquid solution
7	Use	Carbon nanotube	Carbon nanotube	40	100	1	1	Powder and liquid solution
		Silicon dioxide	Silicon dioxide	100	50,000	1	1	Powder
8	Use	Carbon nanotube	Carbon nanotube	0.5	Commercial secret	Commercial secret	Commercial secret	Liquid solution
9	Use	Silicon dioxide	Silicon dioxide	12–17	300	0.2	4	Powder
10	Mfg	Silicon dioxide		160	2500	5	1	Liquid solution
		Silicon dioxide	Silicon dioxide	100–200	60,000	5	8	Colloid
11	Use	Carbon nanotube	Carbon nanotube	110	5000	0.5	4.5	Powder, liquid solution, and gel
	Mfg	Carbon nanotube	Carbon nanotube	110	4,000,000	4	12	Powder and liquid solution
12	Use	Carbon nanotube	Carbon nanotube	100	>20	1	3.5	Powder
		Nano-silver		>100	>40	1	3.5	Liquid solution
13	Use	Titanium dioxide	Titanium dioxide	Unknown	5	6	0.33	Liquid solution

Abbreviation: mfg: manufacturing; mfd: manufactured.

Table S3. Information on the duration and frequency of nanomaterials used, personal protective equipment and ventilation system among the nanomaterials exposed group.

	**NM exposed group (*n* = 241)**	
Nanomaterials used	Mean	(SD)
Frequency of operation (times/week)	2.90	(1.92)
Duration of operation (hours/time)	3.01	(3.26)
Years of exposure (year)	2.96	(2.14)
Total exposure time (hours)	1495	(3271)
	n	(%)
Nanomaterials category		
Carbon nanotube	57	(23.7)
Nano-Tio_2	17	(7.1)
Nano-Sio_2	36	(14.9)
Nano-Ag	16	(6.6)
Other nanomaterial exposure (Nano resin, Nan clays, and Nano-Au)	54	(22.4)
Over two types of nanomaterials exposure	61	(25.3)
Ventilation system		
General ventilation systems	41	(17.0)
Local exhaust system	114	(47.3)
Industrial fan	20	(8.3)
Others	66	(27.4)
Protective equipment		
No	19	(7.9)
Cotton mask	16	(6.6)
Activated carbon mask	58	(24.1)
N95 medical mask	9	(3.7)
Gas mask	15	(6.2)
Others	124	(51.5)

Table S4. Summary of the most important characteristics of probability scores.

**Variables/scores**	**30 pts**	**25 pts**	**22.5 pts**	**18.75 pts**	**15 pts**	**12.5 pts**	**11.25 pts**	**10 pts**	**7.5 pts**	**6.25 pts**	**5 pts**	**0 pts**
Estimated amount of material used	-	>100 mg	-	Unknown	-	11–100 mg	-	-	-	0–10 mg	-	-
Dustiness/mistiness	High	-	Unknown	-	Medium	-	-	-	Low	-	-	None
Number of employees with similar exposure	-	-	-	-	>15	-	Unknown	11–15	-	-	6–10	1–5
Frequency of operation	-	-	-	-	Daily	-	-	Weekly	-	-	Monthly	Less than monthly
Duration of operation	-	-	-	-	>4 h	-	Unknown	1–4 h	-	-	30–60 min	<30 min

Table S5. Summary of the most important characteristics of severity factors and scores.

**Variables/scores**	**10 pts**	**7.5 pts**	**6 pts**	**5 pts**	**4.5 pts**	**4 pts**	**3 pts**	**2.5 pts**	**0 pts**
Nanomaterial									
Surface chemistry	High	Unknown	-	Medium	-		-		Low
Particle Shape	Tubular/fibrous	Unknown	-	Anisotropic	-		-		Compact/Spherical
Particle Diameter	1–10 nm	Unknown	-	11–40 nm	-		-		>41 nm
Solubility	Insoluble	Unknown	-	soluble	-		-		-
Carcinogenicity	-	-	Yes	-	Unknown		-		No
Reproductive Toxicity	-	-	Yes	-	Unknown		-		No
Mutagenicity	-	-	Yes	-	Unknown		-		No
Dermal Toxicity	-	-	Yes	-	Unknown		-		No
Asthmagen	-	-	Yes	-	Unknown		-		No
Parent Material									
Occupational exposure limit	<10 $\mu g/m^3$	Unknown	-	10–100 $\mu g/m^3$	-		-	101–1000 $\mu g/m^3$	-
Carcinogenicity	-	-	-	-		Yes	Unknown	-	No
Reproductive Toxicity	-	-	-	-		Yes	Unknown	-	No
Mutagenicity	-	-	-	-		Yes	Unknown	-	No
Dermal Toxicity	-	-	-	-		Yes	Unknown	-	No

Table S6. The classification of the different NPs in term of Risk levels.

Variables	**RL1 (*n* = 126)**		**RL2 (*n* = 115)**		
	n	(%)	*n*	(%)	*p*-Value [a]
Carbon nanotube	25	(19.8)	32	(27.8)	0.090
Nano-TiO_2	9	(7.1)	8	(7.0)	
Nano-SiO_2	23	(18.3)	13	(11.3)	
Nano-Ag	10	(7.9)	6	(5.2)	
Other NM exposure	34	(27.0)	20	(17.4)	
More than two types of NM exposure	25	(19.8)	36	(31.3)	

[a] Chi-Square Tests.

© 2014 by the authors; licensee MDPI, Basel, Switzerland. This article is an open access article distributed under the terms and conditions of the Creative Commons Attribution license (http://creativecommons.org/licenses/by/3.0/).
